# Axitinib plus immune checkpoint inhibitor: evidence- and expert-based consensus recommendation for treatment optimisation and management of related adverse events

**DOI:** 10.1038/s41416-020-0949-9

**Published:** 2020-06-26

**Authors:** Viktor Grünwald, Martin H. Voss, Brian I. Rini, Thomas Powles, Laurence Albiges, Rachel H. Giles, Eric Jonasch

**Affiliations:** 1 Interdisciplinary GU Oncology, West German Cancer Center Essen, Clinic for Urology and Clinic for Medical Oncology, University Hospital Essen, Essen, Germany; 2 Memorial Sloan Kettering Cancer Center, New York, NY USA; 3 Cleveland Clinic Taussig Cancer Institute, Cleveland, OH USA; 4 Barts Cancer Institute, Queen Mary University of London, London, UK; 5 Gustave Roussy Institute, Université Paris Saclay, Villejuif, France; 6 International Kidney Cancer Coalition, Duivendrecht, The Netherlands; 7 The University of Texas MD Anderson Cancer Center, Houston, TX USA

**Keywords:** Renal cell carcinoma, Renal cancer

## Abstract

With the recent approval of the combinations of axitinib with the immune checkpoint inhibitor (ICI) pembrolizumab or avelumab for first-line treatment of advanced renal cell carcinoma, guidance on how to distinguish between immune-related adverse events (AEs) caused by ICI versus axitinib-related AEs is necessary to optimise therapy with axitinib–ICI combinations. The recommendations here are based on (1) systematic review of published evidence, (2) discussion among experts in the field and (3) a survey to obtain expert consensus on specific measures for therapy management with the combinations axitinib/avelumab and axitinib/pembrolizumab. The experts identified areas of AEs requiring unique management during treatment with axitinib–ICI combinations that were not covered by current recommendations. Diarrhoea, hepatic toxicity, fatigue and cardiovascular AEs were found to be applicable to such specialised management. Triage between immune-suppressive and supportive measures is a key component in therapy management. Clinical monitoring and experience with both classes of agents are necessary to manage this novel therapeutic approach. We focused on AEs with an overlap between axitinib and ICI therapy. Our recommendations address AE management of axitinib–ICI combinations with the aim to improve the safety of these therapies.

## Background

Tyrosine kinase inhibitors (TKIs) targeting the vascular endothelial growth factor (VEGF) pathway improved outcomes in patients with metastatic renal cell carcinoma (mRCC), but most patients develop resistance to these antiangiogenic agents.^1–6^ Immune checkpoint inhibitors (ICIs) targeting the programmed cell death-1 (PD-1) receptor or its ligand (PD-L1) produced durable responses in different types of tumours, including mRCC.^7–9^

Studies have shown that, in addition to their antiangiogenic activity, inhibitors of the VEGF pathway enhance the antitumour activity of ICIs by blocking tumour-induced immune-suppressive cells and increasing T-cell infiltration into tumours.^10–12^ The results of two phase III trials of the combinations of axitinib (a selective inhibitor of VEGF receptors 1–3) plus avelumab (anti-PD-L1) or pembrolizumab (anti-PD-1) versus sunitinib have been recently published.^13,14^

In the JAVELIN Renal 101 trial, median (95% confidence interval [CI]) progression-free survival (PFS) in the overall population was 13.8 (11.1—not estimable) months with axitinib/avelumab and 8.4 (6.9–11.1) months with sunitinib (hazard ratio [HR] 0.69 [95% CI: 0.56–0.84, *P* = 0.0001]); the median follow-up time was 10.8 months and 8.6 months, respectively.^13^ Median overall survival (OS) at interim analysis was HR 0.78 (95% CI: 0.55–1.08) and objective response rate (ORR) was 51% with axitinib/avelumab and 26% with sunitinib.^13^ The final OS analysis is awaited. In the KEYNOTE-426 trial, median (95% CI) PFS in the overall population was 15.1 (12.6–17.7) months with axitinib/pembrolizumab and 11.1 (8.7–12.5) months with sunitinib (HR 0.69, 95% CI: 0.57–0.84, *P* < 0.001); the median follow-up time was 12.8 months. HR for OS was 0.53 (95% CI: 0.38–0.74). The ORR was 59% with axitinib/pembrolizumab and 36% with sunitinib.^14^

Based on the outcomes of these studies, the US Food and Drug Administration (FDA) and European Medicines Agency recently approved axitinib in combination with avelumab or pembrolizumab for the first-line treatment of patients with advanced RCC.^15–17^

Therapy with ICIs is associated with distinct immune-related adverse events (AEs) often caused by a nonspecific activation of the immune system, and can impact multiple organ systems. Toxicity management includes administration of immunosuppressants such as systemic corticosteroids. In contrast, toxicities from antiangiogenic agents are composed of a variety of different underlying mechanisms,^18^ which are thought to be predominantly not immune-mediated, and are primarily managed by treatment interruption and dose reductions. The use of ICIs in combination with antiangiogenic agents adds complexity to the management of toxicity, as some of the AEs seen with ICIs are similar to those typically seen with antiangiogenic therapy. Recognising the aetiology of each AE is important but not always possible.

Several guidelines have been published on management of toxicities associated with ICIs or antiangiogenic therapy.^19–21^ However, there is an unmet need for guidelines on differential management of AEs derived from ICI or antiangiogenic therapy. With the approval of the combinations axitinib/avelumab and axitinib/pembrolizumab for mRCC treatment, the goal of this paper is to offer guidance to clinicians on how to optimise treatment and manage toxicities associated with TKI–ICI combination therapy. A particular emphasis includes how to distinguish between immune-related AEs caused by avelumab or pembrolizumab versus AEs resulting from axitinib when combined with avelumab or pembrolizumab.

## Methods

### Assembly of experts in RCC

Clinical experts with background in medical treatment in RCC and a patient representative were convened for a virtual meeting on 28 June 2019 to discuss the need for differential therapy management guidelines and received honoraria from Pfizer for their participation as advisers. All of the advisers who attended the meeting are named authors of this paper. Support for the literature analysis and medical writing was funded by Pfizer and Merck KGaA. The authors retained full control over the content of the paper.

### Search strategy and selection criteria

We conducted a systematic review of the literature, using PubMed, for any publications on axitinib/avelumab and axitinib/pembrolizumab combinations in the first-line setting. In addition, the following congresses were hand-searched for abstracts: American Society of Clinical Oncology (ASCO) Annual Meeting 2018, European Society of Medical Oncology (ESMO) Congress 2018, Genitourinary Cancers Symposium 2019 and ASCO Annual Meeting 2019. The Preferred Reporting Items for Systematic Reviews and Meta-Analyses (PRISMA) guidelines were followed.^22,23^ The following search terms were used: “carcinoma”, “malignant”, “tumor”, “tumour”, “neoplas” and “cancer”, in combination with “first line”, “first-line”, “front-line”, “front line”, “frontline”, “naïve”, “untreated”, “avelumab and axitinib” and “avelumab plus axitinib” and “pembrolizumab and axitinib” and “pembrolizumab plus axitinib”.

Studies meeting the following population, intervention, comparator, outcomes and study types were included in this systematic review: (1) population: patients ≥18 years with treatment-naive cancer in first-line setting, (2) intervention/comparator: axitinib plus avelumab and axitinib plus pembrolizumab, (3) outcomes: included but not restricted to AEs, serious AEs, treatment-related AEs, immune-related AEs, treatment duration and discontinuation due to AEs and (4) study type: clinical trials.

Studies were excluded if they were conducted in patients who had prior systemic therapy or did not report safety outcomes, reviews, case reports, editorials, letters or opinions, and papers not published in English were also excluded.

### Data analysis

AEs associated with each combination were compared with the monotherapy treatments (in similar indications) to identify the most commonly occurring (>10%) AEs that may be related to axitinib and/or ICI treatment. Of the AEs identified, advisers determined the AEs that required additional guidance based on overlapping toxicities and lack of proper clinical discrimination. Early signs and symptoms that may lead to serious complications were also reviewed.

Guidance was based on research of published evidence, thorough discussion and synthesis by expert consensus on specific measures for therapy management. The advisers and a patient representative reviewed the currently available guidelines (ASCO, Society for Immunotherapy of Cancer [SITC]) on management of toxicities associated with ICIs and antiangiogenic agents,^20,21^ and indicated gaps in therapy management. The recommendations on therapy management (class IV evidence) were based on the current guidelines (ASCO, European Association of Urology [EAU], ESMO and SITC)^19–21,24^ along with gaps in the guidelines, expert opinion and personal experience with axitinib–ICI combination. The recommendations discussed by the advisers were summarised in a questionnaire that was sent to all advisers after the meeting. The advisers had to respond to each recommendation with “agree” or “disagree”. If “disagree” was selected, the adviser was asked to provide a reason. Recommendations that were approved by at least 70% of the advisers were considered to represent a consensus and were summarised in this report. For the recommendations that were not agreed upon by all advisers, additional discussions (via email) took place until all advisers agreed to the edited recommendations included in this paper.

## Results

### Search results

The PubMed search identified 11 papers and a search of congress abstracts identified nine records. After removal of duplicates, six records (four papers and two abstracts) met the inclusion/exclusion criteria and are listed in Supplementary Table S1.

The most common (>10%) treatment-related AEs associated with each combination that may be immune-related or associated with axitinib monotherapy included endocrine (hypothyroidism and hyperthyroidism), dermatologic (rash/inflammatory dermatitis and pruritus), gastrointestinal (colitis [diarrhoea], hepatic signals [alanine aminotransferase (ALT) and/or aspartate aminotransferase (AST) increased] and nausea), musculoskeletal (inflammatory arthritis [arthralgia]) and general disorders and administration-site conditions (fatigue and infusion-related reaction, Supplementary Table S2).

The currently available guidelines (i.e., ASCO, EAU, ESMO and SITC)^19–21^ provide sufficient guidance, and no further description is required for hypothyroidism, hyperthyroidism, rash/inflammatory dermatitis, pruritus and arthralgia (Supplementary Table S2). Rare AEs of potential interest were also identified, including myasthenia gravis, myocarditis, necrotising fasciitis, pneumonitis, necrotising pancreatitis, cephalalgia and abdominal distension. For these rare AEs, which are mostly seen with ICIs and not TKIs, it is recommended to follow the currently available guidelines.^19–21^

### Treatment recommendations

The recommendations herein focus on AEs that require a differential diagnosis of aetiology (i.e., immune-related vs. axitinib-related AEs), and for which there are currently no published guidelines specific to treatments with axitinib–ICI (Supplementary Table S2). Clinical judgement should be exercised while managing AEs, with up- or downranking of AE grade based on patient’s overall clinical status.

### Diarrhoea

Diarrhoea was commonly reported to be related to patients treated with axitinib monotherapy (45%) and in patients treated with combinations of axitinib/avelumab (54%, Supplementary Table S3) and axitinib/pembrolizumab (49%).^2,13,14,25,26^ Diarrhoea was less common in patients treated with monotherapy avelumab (13%) or pembrolizumab (19%, Supplementary Table S3).^7,9^ For the combinations axitinib/avelumab and axitinib/pembrolizumab, immune-related diarrhoea was reported in 9 (2%) and 11 (3%) patients, respectively.^13,14^ Immune-related colitis was reported in 4 (1%) patients treated with axitinib/avelumab. Based on the incidence of immune-related diarrhoea with single-agent ICIs, a higher frequency is anticipated with the combined treatments. The exact aetiology of diarrhoea with these two classes of drugs remains unknown, although aspects of the clinical presentation may be distinct with VEGF-targeted therapy being associated with a slower onset of symptoms, and lack of fever and abdominal pain.

**Fig. 1:**
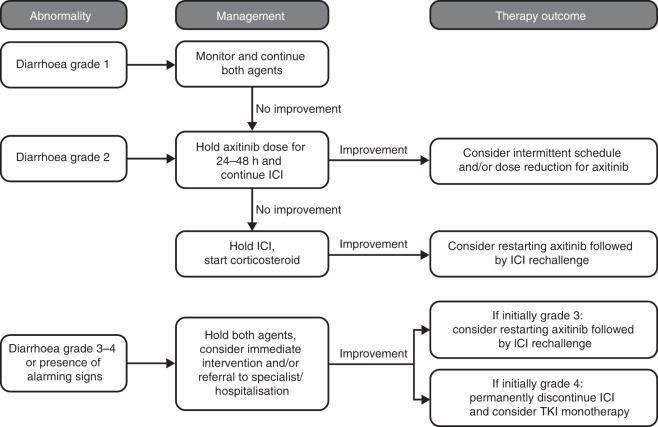
Managing treatment-induced diarrhoea. This flow chart presents our recommended process for managing diarrhoea induced by treatment with the combination of axitinib and ICIs. Supportive care should be provided for all grades. ICI immune checkpoint inhibitor, TKI tyrosine kinase inhibitor. Intermittent schedule: breaks of 24–48 h from axitinib.

A flow chart describing how to manage diarrhoea induced by treatment with the combination of axitinib and ICIs is presented in Fig. 1. It is first recommended to assess the patient’s condition and general state, and to look for alarming clinical signs (e.g., bloody stools, dehydration, fever, sudden onset of watery diarrhoea with rapidly worsening frequency or clinical deterioration), which should trigger immediate intervention and/or referral to a specialist/hospital. Immune-related colitis, especially at higher grades, requires treatment with corticosteroids. In this paper, we use prednisolone as an example, but other equivalent corticosteroids may be used (e.g., prednisone and methylprednisolone). In patients in whom the clinical index of suspicion for immune-related toxicity is high and in those with clinically alarming signs, empiric treatment with prednisolone (1–2 mg/kg) should be initiated immediately, before disclosure of confirmatory test results. Endoscopic evaluation may be considered at the time of the first occurrence and should be attempted in all patients with refractory diarrhoea. Measuring baseline levels of C-reactive protein, stool lactoferrin and/or calprotectin can help identify patients who are experiencing immune-related colitis.^27,28^ While the first occurrence of diarrhoea on therapy is often treatment-associated, other causes may be considered. Previous antibiotics or use of immune-suppressive agents increase the risk for infectious causes of diarrhoea, which should be considered during the process of clinical diagnosis. Hence, assessment of *Clostridium difficile*, ova and parasite, cytomegalovirus or other viral aetiologies should also be considered.

In the absence of alarming signs, axitinib dosing should be withheld for 24–48 h, and the patient should be monitored closely for clinical outcomes. The short half-life of axitinib in the plasma (2.5– 6.1 h) allows for a quick decrease in plasma concentrations after withholding the drug and, as a result, quick recovery from axitinib-related AEs.^29^ If the diarrhoea is axitinib-induced, the symptoms should be partially or completely better within 48 h and, if not, it could potentially be immune-related. If there is improvement (i.e., a decrease in diarrhoea) after withholding axitinib and monitoring for 48 h, consider optimisation of supportive measures, intermittent dosing (e.g., breaks of 24–48 h from axitinib as needed for diarrhoea) and/or axitinib dose reduction. The duration of periodic breaks should be driven by severity, and by relief and reoccurrence upon breaks. In principle, intermittent dosing allows maintenance of a higher axitinib dose compared with dose reduction. Breaks should consider time to occurrence/resolution of diarrhoea, and should be individualised to the patient’s needs, rather than a fixed, discontinuous schedule for all patients.

If there is no improvement after withholding axitinib and monitoring for 48 h, assume immune-related diarrhoea and follow existing guidelines.^20,21,24^ For grade 1–2 axitinib-related diarrhoea, consider continuing therapy with dietary adjustment and/or antidiarrhoeal medication supplementation. Axitinib treatment can be withheld or reduced due to persistent, low-grade diarrhoea. Patients whose diarrhoea cannot be managed despite dietary adjustment and antidiarrhoeal medication, and who present with signs and symptoms of dehydration, should be considered for extended diagnostics; referral to a specialist/hospitalisation may be considered, regardless of the grade of the event. In cases of bloody stools, high-volume watery stools or sudden onset of severe diarrhoea, immediate action is required. Corticosteroid treatment should be initiated, along with hospitalisation and referral to specialist care if appropriate.

### Hepatitis

Increased transaminases are considered diagnostic for hepatic toxicity, and may have multiple causes, including immune toxicity. Progression of liver metastases is of principal interest in the differential diagnosis, but the incidence of primary treatment failure with axitinib plus ICI remains a rare event (10.5–11.5%).^13,14^ De novo occurrence of elevated transaminases during treatment in the absence of new concomitant medication or other potential hepatotoxins is highly suspicious for treatment-related toxicity and should be treated as such, without delay. On the contrary, in patients with previous exposure to corticosteroids, tests for infectious causes should be included in the diagnostic algorithm. Activation of viral pathogens should be considered in patients at risk (i.e., previous exposure to immunosuppressant medications), and anti-hepatitis A virus immunoglobulin (Ig) M, hepatitis B surface antigen (HBsAg), anti-HBsAg, anti-hepatitis B core antigen IgM and anti-hepatitis C virus (HCV) Ig should be assayed. In the case of positive HCV results, HCV RNA should be assayed.

The incidence of treatment-related ALT and AST increases, respectively, was reported in 9% and 6% of patients treated with axitinib monotherapy, in 13% and 11% of patients treated with axitinib/avelumab and in 24% and 23% of patients treated with combinations of axitinib/pembrolizumab (Supplementary Table S3).^2,13,14^ The FDA label of the combinations axitinib/avelumab and axitinib/pembrolizumab includes special warnings for hepatic toxicity and hepatitis.^30,31^

**Fig. 2:**
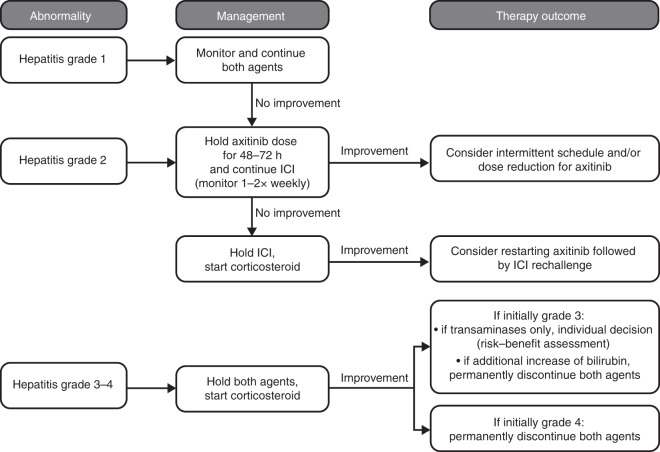
Managing treatment-induced hepatitis. This flow chart presents our recommended process for managing hepatitis induced by treatment with the combination of axitinib and ICIs. Supportive care should be provided for all grades. Concomitant bilirubin elevation with any grade should prompt full workup, stopping both drugs and referral to hepatology. ICI immune checkpoint inhibitor. Intermittent schedule: breaks of 48–72 h from axitinib.

A flow chart describing how to manage axitinib plus ICI treatment-induced hepatitis is presented in Fig. 2. It is first recommended that the patient’s condition and general state is assessed. For grade 1–2 hepatitis, withhold axitinib and monitor closely for symptoms for 48–72 h before making a final judgement on whether it is axitinib or immune-related. Liver function tests should then be rechecked. If there is improvement of hepatitis after withholding axitinib, consider further treatment interruptions and axitinib dose reduction as described previously for diarrhoea.

**Fig. 3:**
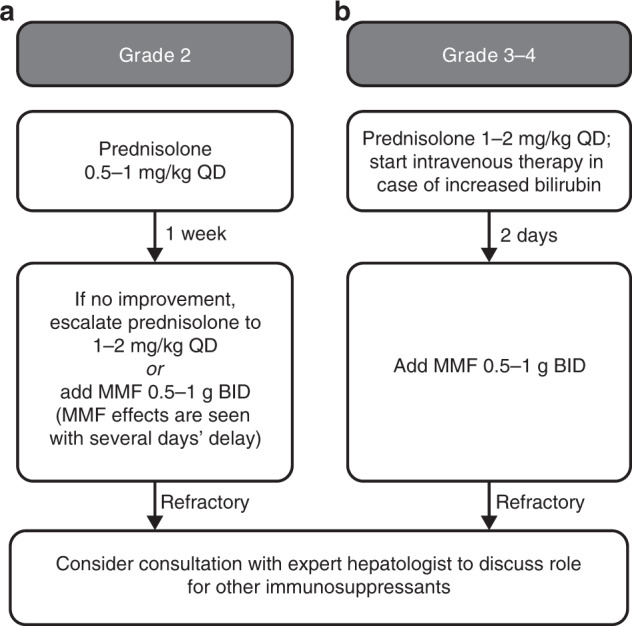
Escalation schema for immunosuppressive treatment of immune-related hepatic toxicity. **a** For grade 2, consider low-dose prednisolone (0.5 mg/kg). In the absence of adequate response within 1 week, escalation of prednisolone to doses of 1–2 mg/kg or addition of MMF should commence. **b** For grade 3–4 hepatic toxicity, high-dose prednisolone (1–2 mg/kg QD) should be considered and escalated by the addition of MMF (BID) within 48 h in the case of inadequate response. In refractory cases, expert consultation is advised. Supportive care should be provided for all grades. BID twice daily, ICI immune checkpoint inhibitor, MMF mycophenolate mofetil, QD once daily.

If there is no clinical improvement after withholding axitinib, both drugs should be stopped, and corticosteroid treatment considered. Worsening hepatitis after withholding axitinib while continuing avelumab or pembrolizumab indicates that the ICI agents are related to the AE; in these cases, corticosteroid treatment should be initiated. For grade 2 immune-related transaminitis, low-dose prednisolone (0.5 mg/kg) is recommended, with twice-weekly monitoring of liver function tests. If no improvement is seen within a 2-week window, addition of mycophenolate mofetil (MMF) 0.5–1 g twice daily is recommended (Fig. 3). Because there is an observed delay in the response to MMF treatment (~5–7 days), it is important to consider timely initiation of MMF and maintaining prednisolone use once MMF is started. In the case of inadequate response within 2 weeks, dose escalation of prednisolone to 1–2 mg/kg once daily is mandated. Immunosuppression should be adapted to the clinical needs in the case of clinical deterioration or severe worsening of abnormalities.

In cases with grade 3–4 hepatitis, interruption of both agents and the initiation of high-dose prednisolone 1–2 mg/kg is recommended. For these patients and for any patients with concomitant bilirubin elevation, a full investigation of hepatic function, including imaging and referral to a hepatologist, is indicated.

### Fatigue

Fatigue is a symptom with multidimensional causes, which may include endocrine abnormalities as a reversible cause.^32^ The overlap of fatigue between both treatment modalities and cancer itself implies a diagnostic dilemma. In the absence of endocrine dysfunction and non-axitinib-related fatigue, other reversible causes and cancer-related fatigue should be considered.

Treatment-related fatigue was commonly reported in patients treated with axitinib monotherapy (27%) as well as in those treated with combination axitinib/avelumab (36%) or axitinib/pembrolizumab (30%, Supplementary Table S3).^2,13,14^ Fatigue was not reported as immune-related in the combination phase III trials.^2,13,14^ In the phase I trial of axitinib/avelumab, treatment-related fatigue was reported in 46% of patients, and no cases of fatigue were reported as immune-related.^26^ In the phase I trial of axitinib/pembrolizumab, treatment-related fatigue was reported in 73% of patients and immune-related fatigue in 12%.^25^ Treatment-related fatigue was also common in patients treated with avelumab (18%) or pembrolizumab monotherapies (25%, Supplementary Table S3).^7,9^

**Fig. 4:**
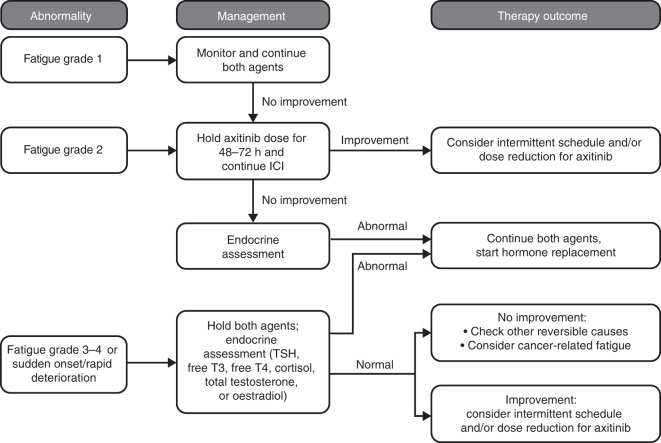
Managing fatigue. This flow chart presents our recommended process for managing fatigue induced by treatment with the combination of axitinib and ICIs. For endocrine assessment, test for TSH, free T3, free T4, cortisol, total testosterone or oestradiol. Supportive care should be provided for all grades. ICI immune checkpoint inhibitor, TSH thyroid-stimulating hormone, T3 triiodothyronine, T4 thyroxine. Intermittent schedule: breaks of 48–72 h from axitinib.

Fatigue can be an early sign of multiple AEs and causalities (e.g., hypothyroidism). Treatment-related hypothyroidism was reported in patients treated with axitinib monotherapy (21%) as well as in those treated with combination axitinib/avelumab (24%) or axitinib/pembrolizumab (32%).^2,13,14^ Proper endocrine surveillance throughout the course of treatment identifies reversible causes of fatigue. Sudden onset, rapid deterioration within a few days, gradual increase in the setting of stable disease, de novo and/or severe cephalgia or de novo visual impairment should trigger investigations of the hormonal axis (thyroid-stimulating hormone, free triiodothyronine, free thyroxine, total testosterone, oestrogen and cortisol). Abnormalities should trigger hormone replacement, and referral to endocrinology should be considered. In any other case, axitinib should be withheld for 48–72 h to assess for improvement before investigating further (Fig. 4). If improvement of fatigue occurs upon stopping axitinib, intermittent treatment or dose reductions for axitinib should be considered.

### Cardiovascular AEs

Cardiovascular AEs occur frequently during treatment with axitinib, which may include hypertension (49%) or chest pain (5%). Immune-related cardiac events are rare (<1%), but have been reported to be fatal.^13,14^ The spectrum of immune-related cardiac events is broad and includes arrhythmias and myocarditis. Myocarditis was reported in 0.06% of patients treated with nivolumab and 0.27% of patients treated with the combination nivolumab/ipilimumab.^33^ Clinical signs of dyspnoea, heart failure or arrhythmias should prompt immediate attention and diagnostic workup, and early referral to a specialist should be considered. Electrocardiogram, troponin I or T, N-terminal pro-brain natriuretic peptide and creatinine kinase are recommended as diagnostics to assess cardiac symptoms. Additional workup may be warranted.

Axitinib-associated hypertension is frequent and may be associated with the cardiovascular events listed previously. Therefore, continuous monitoring and early medical treatment with antihypertensive agents (e.g., calcium channel blockers or angiotensin-converting enzyme inhibitors) is recommended for axitinib-associated hypertension. The objective is to achieve a blood pressure window of 120/80–140/95 mmHg. Treatment often requires antihypertensive combinations, but usually results in sufficient response. Dose reduction of axitinib is rarely needed in order to control hypertension. Notably, holding axitinib for toxicity as noted previously can result in hypotension in patients who had previous intensification of their antihypertensive regimen. Thus, careful monitoring of blood pressure during axitinib holds, and consideration of holding antihypertensive agent(s) concomitantly should be considered.

Cardiac symptoms in the absence of hypertension may be immune-related and should prompt further investigation and cardiologic consultation, as symptoms may be masked. The exact rate of immune-related cardiac toxicities with the combination of axitinib and ICI is currently not known, and proper monitoring is recommended.

Where available, cardiac magnetic resonance imaging may reveal evidence of inflammatory changes, but the role of imaging modalities remains vague, and biopsy should be considered in some patients. Due to the high mortality associated with immune-related myocarditis, aggressive management needs to be initiated with high-dose prednisolone (1–2 mg/kg). Cardiac monitoring in an intensive-care unit should be considered, given the high risk for high-grade conduction delays and ventricular arrhythmias. Additional immunosuppression may be required in severe cases or based on the myocardial biopsy findings, although it is not advisable to delay treatment and wait for the full investigative findings. The decision to treat should be made clinically.

## Conclusions

With the approval of combination axitinib/avelumab and axitinib/pembrolizumab for advanced RCC, the number of patients treated with these combinations in real-world settings is expected to increase. Strategies to distinguish between immune-related AEs caused by avelumab or pembrolizumab versus those resulting from axitinib are necessary to optimise therapy with axitinib–ICI combinations and to support patient quality of life.

Appropriate patient materials and instructions should be provided to facilitate reporting of all AEs. Furthermore, as a general rule for low-grade AEs emerging from treatment with the axitinib/avelumab or axitinib/pembrolizumab combination, it is recommended to first assess the patient for signs and symptoms that require immediate intervention. In the case of severe immune-related toxicities, the treatment decision should be made clinically, thereby avoiding delay of proper treatment. In such cases, diagnostic studies are used to validate the clinical diagnosis and to support initiation of treatment for toxicity. Patients should also be monitored for psychosocial signals that may impact their navigation through a complex treatment causing AEs or impacting their daily life significantly.

In the absence of serious clinical signs, the first intervention should be to withhold axitinib dosing and observe for AE resolution/improvement. If there is improvement in severity of the AE, introduce intermittent treatment and/or dose reduction for axitinib to manage the toxicity accordingly. If no improvement is shown, consider the possibility of an immune-related AE and treat with corticosteroids.

These recommendations are meant to provide a therapeutic window for therapy management of axitinib–ICI treatment. Clinical presentation may be more complex than presented in this paper and should therefore take the complete clinical picture into account. Deviations from recommendations may be warranted and should be based on proper clinical judgement.

The recommendations presented here (class IV evidence) are intended for AEs resulting from treatment with the combination of axitinib/avelumab or axitinib/pembrolizumab. It is unclear if these recommendations may be generalised to other TKI or ICI agents, as differences in pharmacokinetics and AE profiles exist between TKIs. It is important to note that AEs can occur late, even a few months after treatment discontinuation. It is especially relevant to ICI-induced AEs.^20,24^ For late-occurring AEs, it is important to follow the same treatment algorithm as for early-occurring AEs, but further investigation into the causes beyond the combination therapy may be required. It is essential to inform patients about the possibilities of late-occurring AEs.

The recommendations provided in this report represent the consensus agreement achieved by experts in the field, including a patient representative. These recommendations are based on evidence from published literature and personal clinical experience with axitinib–ICI combinations. Implementation of these recommendations may improve the safety of axitinib–ICI combination therapy, and help keep patients on treatment with the goal to achieve better treatment outcomes. These recommendations will be reviewed and potentially updated as more clinical experience with the new combinations is gathered.

## Supplementary information

Axitinib plus immune checkpoint inhibitor: evidence- and expert-based consensus recommendation for treatment optimisation and management of related adverse events

## Data Availability

Not applicable.
