# Supplementary material for: Axitinib plus immune checkpoint inhibitor: evidence- and expert-based consensus recommendation for treatment optimisation and management of related adverse events
Source: Br J Cancer. 2020 Jun 26;123(6):898–904. doi: 10.1038/s41416-020-0949-9 (PMC7492460; doi:10.1038/s41416-020-0949-9)
Supplement: Supplementary file 1 — Axitinib plus immune checkpoint inhibitor: evidence- and expert-based consensus recommendation for treatment optimisation and management of related adverse events [file 41416_2020_949_MOESM1_ESM.docx]

**SUPPLEMENTARY INFORMATION**

*Title:* **Axitinib plus immune checkpoint inhibitor: evidence- and expert-based consensus recommendation for treatment optimisation and management of related adverse events**

*Authors:* Viktor Grünwald, Martin H. Voss, Brian I. Rini, Thomas Powles, Laurence Albiges, Rachel H. Giles and Eric Jonasch

**Supplementary Table S1.** Studies conducted with the axitinib/pembrolizumab or axitinib/avelumab combinations.

**Supplementary Table S2.** Most common treatment-related adverse (>10%) event with axitinib-ICI combinations and guidelines availability.

**Supplementary Table S3.** Incidence of treatment-related adverse events with single ICI and with axitinib-ICI combinations.

**Supplementary Table S1.** Studies conducted with the axitinib/pembrolizumab or axitinib/avelumab combinations.

| **Citation** |
| --- |
| ***Papers*** |
| Atkins MB, *et al*. Axitinib in combination with pembrolizumab in patients with advanced renal cell cancer: a non-randomised, open-label, dose-finding, and dose-expansion phase 1b trial. *Lancet Oncol* 2018;19:405–415. |
| Choueiri TK, *et al*. Preliminary results for avelumab plus axitinib as first-line therapy in patients with advanced clear-cell renal-cell carcinoma (JAVELIN Renal 100): an open-label, dose-finding and dose-expansion, phase 1b trial. *Lancet Oncol* 2018;19:451–460. |
| Rini BI, *et al*. Pembrolizumab plus axitinib versus sunitinib for advanced renal-cell carcinoma. *N Engl J Med* 2019;380:1116–1127. |
| Motzer RJ, *et al*. Avelumab plus axitinib versus sunitinib for advanced renal-cell carcinoma. *N Engl J Med* 2019;380:1103–1115. |
| ***Abstracts*** |
| Kudo M, *et al*. First-line avelumab + axitinib in patients with advanced hepatocellular carcinoma: results from a phase 1b trial (VEGF Liver 100). *J Clin Oncol* 2019;37(Suppl 15):4072 (abstract). |
| Neyns B, *et al*. GLIAVAX: a stratified phase II clinical trial of avelumab and axitinib in patients with recurrent glioblastoma. *J Clin Oncol* 2019;37(Suppl 15):2034 (abstract). |

**Supplementary Table S2.** Most common treatment-related adverse (>10%) event with axitinib-ICI combinations and guidelines availability.

| **Adverse event** | **Currently available guidelines apply** | **Specific recommendation provided herein** |
| --- | --- | --- |
| Hypothyroidism | ✓ |  |
| Hyperthyroidism | ✓ |  |
| Rash/inflammatory dermatitis | ✓ |  |
| Pruritus | ✓ |  |
| Diarrhoea |  | ✓ |
| Hepatitis |  | ✓ |
| Alanine aminotransferase increased |  | ✓ |
| Aspartate aminotransferase increased |  | ✓ |
| Nausea | ✓ |  |
| Inflammatory arthritis (arthralgia) | ✓ |  |
| Fatigue |  | ✓ |
| Infusion-related reaction | ✓ |  |

*ICI* immune checkpoint inhibitor

**Supplementary Table S3.** Incidence of treatment-related adverse events with single ICI and with axitinib-ICI combinations.

|  | Avelumab^1^  (*N* = 62) | Pembrolizumab^2^  (*N* = 110) | Axitinib^3^  (*N* = 189) | Axitinib/pembrolizumab^4^  (*N* = 429) | Axitinib/avelumab^5^  (*N* = 434) |
| --- | --- | --- | --- | --- | --- |
| **Diarrhoea (%)** | | | | | |
| All grades | 13 | 19 | 45 | 49 | 54 |
| Grade 3–4 | 0 | 4 | 9 | 7 | 5 |
| **Alanine aminotransferase increased (%)** | | | | | |
| All grades | NA | 7 | 9 | 24 | 13 |
| Grade 3–4 | NA | NA | NA | 12 | 5 |
| **Aspartate aminotransferase increased (%)** | | | | | |
| All grades | NA | 7 | 6 | 23 | 11 |
| Grade 3–4 | NA | 2 | NA | 7 | 3 |
| **Fatigue (%)** | | | | | |
| All grades | 18 | 25 | 27 | 30 | 36 |
| Grade 3–4 | 0 | NA | NA | 2 | 3 |

*ICI* immune checkpoint inhibitor, *NA* not available

**References**

1. Vaishampayan UN, *et al. J Immunother Cancer* 2019;7:275.
2. McDermott DG, *et al. J Clin Oncol* 2019;37(Suppl 7):546.
3. Hutson TE, *et al.* *Lancet Oncol* 2013;14:1287–1294.
4. Rini BI, *et al. N Engl J Med* 2019;380:1116–1127.
5. Motzer RJ, *et al. N Engl J Med* 2019;380:1103–1115.
